# Immunosuppressive Effects of Multipotent Mesenchymal Stromal Cells on Graft-Versus-Host Disease in Rats Following Allogeneic Bone Marrow Transplantation

**DOI:** 10.4274/Tjh.2013.0032

**Published:** 2013-09-05

**Authors:** Oral Nevruz, Ferit Avcu, A. Uğur Ural, Aysel Pekel, Bahar Dirican, Mükerrem Safalı, Elvin Akdağ, Murat Beyzadeoğlu, Tayfun İde, Ali Sengül

**Affiliations:** 1 Gülhane Medical Academy, Department of Hematology, Ankara, Turkey; 2 Gülhane Medical Academy, Department of Immunology, Ankara, Turkey; 3 Gülhane Medical Academy, Department of Radiation Oncology, Ankara, Turkey; 4 Gülhane Medical Academy, Department of Pathology, Ankara, Turkey; 5 Gülhane Medical Academy, Department of Medical Oncology Research Center, Ankara, Turkey

**Keywords:** Mezenchimal stromal cell, Bone marrow transplantation, İmmunsupresion

## Abstract

**Objective:** Graft-versus-host disease (GVHD) is a major obstacle to successful allogeneic bone marrow transplantation (allo-BMT). While multipotent mesenchymal stromal cells (MSCs) demonstrate alloresponse in vitro and in vivo, they also have clinical applications toward prevention or treatment of GVHD. The aim of this study was to investigate the ability of MSCs to prevent or treat GVHD in a rat BMT model.

**Materials and Methods:** The GVHD model was established by transplantation of Sprague Dawley rats’ bone marrow and spleen cells into lethally irradiated (950 cGy) SDxWistar rat recipients. A total of 49 rats were randomly assigned to 4 study and 3 control groups administered different GVHD prophylactic regimens including MSCs. After transplantation, clinical GVHD scores and survival status were monitored.

**Results:** All irradiated and untreated control mice with GVHD died. MSCs inhibited lethal GVHD as efficiently as the standard GVHD prophylactic regimen. The gross and histopathological findings of GVHD and the ratio of CD4/CD8 expression decreased. The subgroup given MSCs displayed higher in vivo proportions of CD25+ T cells and plasma interleukin-2 levels as compared to conventional GVHD treatment after allo-BMT.

**Conclusion: **Our results suggest that clinical use of MSCs in both prophylaxis against and treatment of established GVHD is effective. This study supports the use of MSCs in the prophylaxis and treatment of GVHD after allo-BMT; however, large scale studies are needed.

**Conflict of interest:**None declared.

## Abstract (tr)

**Amaç:** Graft versus host hastalığı (GVHH) , başarılı bir kemik iliği nakli için önemli bir engel oluşturmaktadır. Multipotent mezenşimal stromal hücrelerin (MSH) immünsupresif etkileri, in vivo ve in vitro olarak gösterilmiş olmakla birlikte, GVHH’ nı önleme yönünde klinik uygulamalarda bulunmaktadır .

**Gereç ve Yöntemler:** Bu çalışmanın amacı ratlarda kemik iliği nakli sonrası oluşturulan GVHH’nı önleme ve tedavi etmede MSH nin etkinliğinin incelenmesidir. Bu amaçla 49 Sprague Dawley cinsi rat rastegele 4 çalışma, 3 kontrol grubuna ayrılmış ve gruplara MSH de içeren farklı GVHH önleyici tedaviler uygulanmıştır. Kemik iliği nakli sonrası GVHH skorlaması ve yaşama süreleri incelenmiştir.

**Bulgular:** Tüm ışınlanmış ve önleyici tedavi verilmemiş ratlar ölmüştür. MSH nin önleyici uygulamaları, standart GVHD önleyici tedavileri kadar etkin bulunmuştur. MSH uygulamaları, GVHH nın gözlemsel ve histolojik bulgularını ve CD4+/CD8+ oranını azaltmaktadır.Ayrıca MSH uygulanan gruplarda CD25+ T hücrelerinin in vivo oranıda daha yüksek olup, Allojeneik kemik iliği nakli sonrası standart GVHH tedavisi uygulananlara göre plazma İnterlökin-2 seviyesinin daha yüksek olarak saptanmıştır.

**Sonuç:** Bulgularımız MSH uygulamasının, GVHH nın hem önlenme hem de tedavi edilmesinde etkin olduğunu göstermiştir. Ancak bu bulguların geniş ölçekli çalışmalarla desteklenmesi gerekmektedir.

## INTRODUCTION

Allogeneic hematopoietic stem cell transplantation (allo-HSCT) after high-dose marrow-ablative chemoradiotherapy is an effective treatment method in various hematologic, neoplastic, and congenital disorders. The major complication after allo-HSCT is the development of graft-versus-host disease (GVHD). GVHD is a life-threatening complication even when the major histocompatibility complex is matched [1,2]. Immunosuppressive therapy (i.e. cyclosporine and/or steroids) is still the first-line treatment for established GVHD; however, the outcome for patients with steroid-resistant acute GVHD is poor, as is overall survival [3]. 

Multipotent mesenchymal stromal cells (MSCs) are multipotent progenitor cells that can differentiate along multiple mesenchymal lineages including bone, cartilage, or fat and expand extensively in vitro [4,5]. The interest in MSC therapy has been raised by the observation that MSCs are able to modulate immune responses in vitro and in vivo [6]. MSCs display immunosuppressive properties that suppress the proliferation of T cells induced by alloantigens or mitogens [7]. Furthermore, MSCs have been reported to induce T cell division arrest, to inhibit the differentiation and maturation of dendritic cells, and to decrease the production of inflammatory cytokines by various immune cell populations [8,9,10]. These properties can be utilized in the context of allo-HSCT, particularly to modulate GVHD and graft rejection [6]. Therefore, MSCs can be thought of as promising agents for severe steroid-resistant acute GVHD and nonresponders can be treated with alternative methods, including MSCs (11). 

The aim of this study was to evaluate the prophylactic and therapeutic potential of MSCs against GVHD using an established rat model of acute GVHD. 

## MATERIALS AND METHODS

**Animals**

Female Wistar rats of 10-12 weeks old were used as recipients and male Sprague Dawley (SD) rats as donors. All procedures were performed according to the institutional guide for animal experimentation and the study protocol was approved by the institutional ethics committee. 

**Bone Marrow Preparation and Bone Marrow-Derived Rat MSC Generation **

Bone Marrow Preparation and Bone Marrow-Derived Rat MSC Generation Briefly, SD rats were sacrificed by decapitation and bone marrow (BM) was flushed with L-DMEM (Gibco, Grand Island, NY, USA) using a 23-gauge needle from femurs and tibias. The BM cells were then pelleted by centrifugation at 1000 rpm for 15 min. The BM cells were gently resuspended using an 18-gauge needle and filtered through a sterile nylon mesh. The viability was consistently >95% as determined by trypan blue exclusion. 

For the MSC generation, BM cells were plated in 25-cm2 polystyrene flasks in L-DMEM supplemented with 10% fetal bovine serum at 37 °C with 5% CO2 conditions (Gibco). Cells were allowed to adhere for 72 h followed by the removal of nonadherent cells and media were changed every 3 to 4 days. Adherent cells were detached using trypsin-EDTA solution-B (EDTA 0.05%, trypsin 0.25%, with phenol red; Biological Industries, Beit-Haemek, Israel) at 37 °C for 10 min and MSCs were expanded 3-4 times to achieve the desired cell numbers for use in in vitro and in vivo experiments. 

**Preparation of Viable Splenocytes**

SD rats weighing between 200 and 250 g were sacrificed by decapitation to be used as donors for splenocytes. After sterile splenectomy, spleens were collected and kept on ice with L-DMEM (Gibco) supplemented with penicillin (100 U/mL) and streptomycin (100 µg/mL). The spleens were disrupted in the medium by pressing spleen fragments between 2 glass slides. Spleen cell suspensions were filtered through a 100-mesh nylon filter and washed 3 times with L-DMEM medium. Splenocyte viability was consistently >95% as determined by trypan blue exclusion. Viable nucleated cells were counted and adjusted to 4.0x10^8^/500 µL. 

**Allo-HSCT Procedure**

We generated a rat GVHD model in the context of allo-HSCT with infusion of 4x108 donor splenocytes. Allo-HSCT was performed with isolation of 1x108 mononuclear cells from male SD rat donors, dilution in 0.5 mL of phosphate-buffered saline, and infusion to female Wistar rat recipients via the tail veins. The recipient animals were conditioned with a myeloablative regimen consisting of 950 cGy of total body irradiation prior to allo-HSCT. The GVHD prophylactic regimen was arranged as 3 mg/kg/day cyclosporine-A (CsA) and 0.25 mg/kg methotrexate (MTX) intraperitoneally at days +1, +3, and +6. Experimental Design A total of 49 Wistar rats were enrolled in this study and they were randomly assigned to 4 study groups (SGs) and 3 control groups (CGs) (n=7 each). The GVHD model was generated in all SGs but only in one CG. 

1. CG-I: Allo-HSCT and GVHD (enforced through donor splenocyte infusion). 

2. CG-II: No allo-HSCT, myeloablative regimen only (total body irradiation). 

3. CG-III: No allo-HSCT, no myeloablative regimen (no GVHD). 

4. SG-I: Allo-HSCT, only standard GVHD prophylactic regimen (CsA+MTX) on day -1 of allo-HSCT. 

5. SG-II: Allo-HSCT, given only MSCs (2x10^6^ cells/kg) on day +1 after allo-HSCT. 

6. SG-III: Allo-HSCT, given standard GVHD prophylactic regimen on day -1 of allo-HSCT plus MSCs (2x106 cells/kg) on day +1 of allo-HSCT. 

7. SG-IV: Allo-HSCT, given MSCs (2x10^6^ cells/kg) after observation of GVHD findings. 

**Figure 1:**
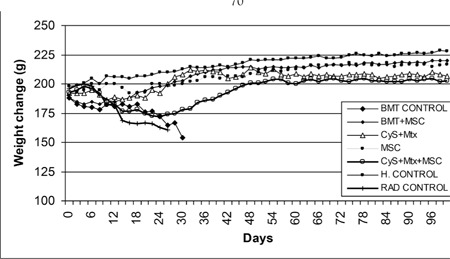
The weight changes of the study and control grouprats during the experiment

**Figure 2:**
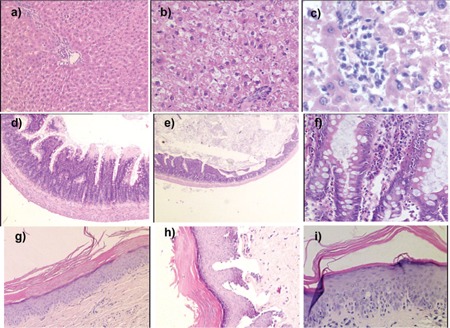
GVHD-related histopathological examinationof groups. a, b, c: Hepatic pathology of GVHD (a: healthycontrol, 50x; b: BMT-control-GVHD, 100x; c: BMT-control-GVHD, 200x). d, e, f: Intestinal pathology of GVHD (d:healthy control, 50x; e: BMT-control-GVHD, 25x; f: BMTcontrol-GVHD, 200x); g, h, i: Dermatological pathologyof GVHD (g: healthy control, 50x; h: BMT-control-GVHD,100x; i: BMT-control-GVHD, 200x).

The rats were observed daily for clinical signs of GVHD, such as diffuse erythematous lesions (particularly of the ears and extremities), hyperkeratosis of the footpads, skin rash, diarrhea, anorexia, and weight loss (Figure 1). Immunophenotypical (CD4, CD8, CD25, and plasma IL-2 levels) examinations and histopathological findings of GVHD (Table 1; Figure 2) following allo-HSCT were performed, and the survival of all groups was monitored. 

**Statistical Analysis**

To evaluate the effects of several variables on overall survival, Kaplan-Meier survival analysis (log-rank statistics) was performed. Differences among the treatment groups were assessed by nonparametric Mann-Whitney U tests. Analyses were managed with SPSS 10.0 (SPSS Inc., Chicago, IL, USA) and the significance level was 5% (p<0.05). 

## RESULTS

**Figure 3:**
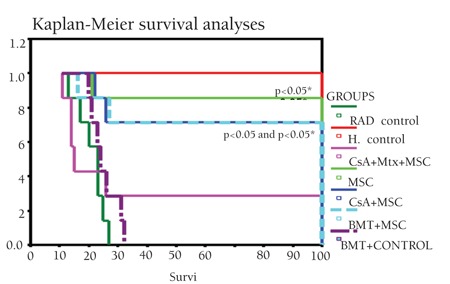
Kaplan-Meier survival curves of the study andcontrol group rats (*: statistical significance in comparisonwith BMT-control).

**Figure 4:**
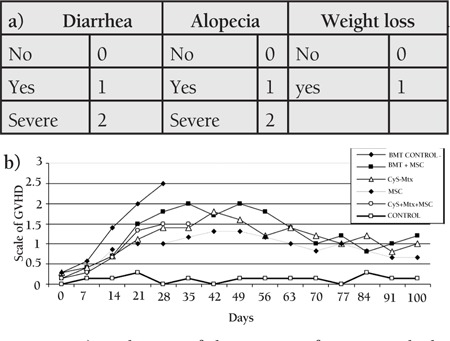
a) Evaluation of the severity of GVHD with thesemiquantitative clinical scoring scale utilizing diarrhea,alopecia, and weight changes over the experiment period;b) degree of GVHD in the study and control group rats

Wistar recipients from male SD donors were transplanted with allogeneic hematopoietic stem cells (allo-HSC) or MSCs as described above. The survival of the Wistar rats as shown in the Kaplan-Meier survival curve following allo-HSCT (Figure 3) was significantly longer in SG-I, SG-II, and SG-IV than in CG-I (p<0.05). However, the survival of SG-III was not significantly longer than in CG-I. The semiquantitative clinical scoring scale showed significant differences of the severity of GVHD (Figure 4). Clinical signs and symptoms of GVHD were significantly lower in SG-I and SG-II than in CG-I (p<0.05 and p<0.05, respectively). However, SG-III and SG-IV were not significantly different from CG-I (p>0.05 and p>0.05, respectively). 

**Figure 5:**
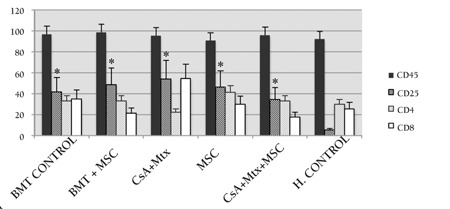
Results of immunophenotypical analyses (CD45,CD4, CD8, CD25) in the study and control groups: CD25was significantly increased (p<0.001) in all study groupsand was higher in CG-I than in CG-III.*: p<0.001 in comparison with healthy control (SG-I).

**Figure 6:**
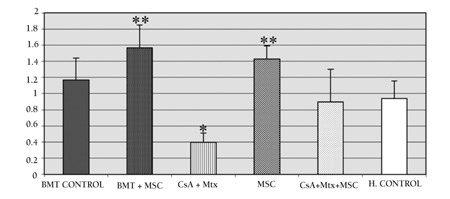
The ratio of CD4/CD8 expression in the studyand control groups. The ratio of CD4/CD8 expressionwas significantly increased in the MSC groups, but ratherdecreased in the CsA+MTX group in comparison with thehealthy control. * p=0.008 in comparison with CG-III (healthy control). ** p=0.014 in comparison with CG-III (healthy control).

**Figure 7:**
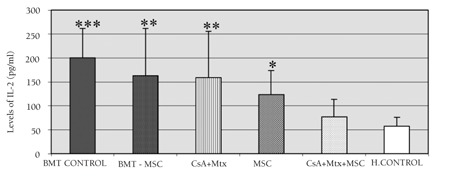
Plasma IL-2 levels (pg/mL) in the study andcontrol groups. Statistical significance in comparison withthe healthy control is shown.* p=0.032 in comparison with CG-III (healthy control).** p=0.018 in comparison with CG-III (healthy control).*** p<0.001 in comparison with CG-III (healthy control).

Statistical evaluation was performed on day 28. Immunophenotypical (CD45, CD4, CD8, and CD25) examinations and plasma IL-2 levels were performed for all groups following allo-HSCT. The expression of CD25 increased significantly (p<0.001) in all SGs and CG-I compared to CG-III. Intergroup comparisons of all SGs and CG-I in terms of CD25 expression were not statistically significant (Figure 5). The ratio of CD4/CD8 expression was significantly lower in SG-I (p=0.008) than in CG-III. In contrast, this expression was significantly higher in SG-II and SG-IV (p=0.014 for both) compared to CG-III (Figure 6). Plasma IL-2 levels were significantly increased in SG-I (p=0.032), SG-II (p=0.018), and SG-IV (p=0.032) compared to CG-I. This increase was much more prominent (p<0.001) in CG-I than in CG-III (Figure 7). 

## DISCUSSION

Allo-HSCT is an increasingly used treatment modality for hematological malignancies [2]. However, GVHD is a life-threatening complication of allo-HSCT caused by donor lymphocytes reacting against host tissues and is a major contributor to morbidity and mortality associated with this procedure [1,12]. Several studies have suggested that MSCs could exert immunomodulatory properties to reduce the incidence of GVHD after allo-HSCT. GVHD can be readily controlled by escalation of systemic immunosuppression (prophylactic regimens or systemic high-dose steroids) in up to 70% of patients [13,14]. However, patients whose GVHD is refractory to this therapy have a poor prognosis. There is no standard second-line or salvage therapy for these patients and various therapeutic modalities have been administered. However, the results so far have demonstrated limited efficacy and low long-term survival due to toxicity [15,16,17,18,19]. 

Administration of MSCs is an alternative option in the treatment of steroid-refractory GVHD. Immunomodulatory properties of MSCs have been exploited to reduce the incidence of GVHD after allo-HSCT [10,11,21,22,23,24,25]. First, the effectiveness of MSCs on GVHD was supported by in vitro studies. Ning et al. first reported the effectiveness of human BM MSCs on allogeneic T lymphocyte phenotype in vitro (26). In vivo studies have also supported the effectiveness of MSCs on GVHD. Tian et al. reported the mechanisms responsible for GVHD in the setting of co-transplantation of allo-HSC and MSCs. They created a model of acute GVHD in rats transplanted with allo-HSC plus donor-derived T cells, with or without additional donor-derived MSC co-transplantation. They suggested that BM-derived MSCs can prevent lethal GVHD following allo-HSCT by means of homeostasis of T lymphocyte subsets in vivo. Their study demonstrated that the value of CD8+ and CD4+ T cells and the ratio of Th1/Th2 T cell subsets decreased at the same time, so the proportion of CD4+, CD25+ T cells increased both in spleen lymphocytes and thymocytes in vivo after allo-HSCT with MSC co-transplantation [27]. Our results, too, suggest that BM-derived MSCs can prevent GVHD after allo-HSCT by means of homeostasis of T subsets in vivo. We also report a decreased ratio of CD4/CD8 expression, along with an increased proportion of CD25+ T cells and plasma interleukin-2 levels in vivo after allo-HSCT with prophylactic MSC administration. We also determined the highest level of CD8 in the CsA+MTX group. The highest level of CD8 in the CsA+MTX group may have an additional vrole in the treatment of GVHD. Recent studies particularly suggested inducible CD8 cells to be useful in suppressing autoimmune reactions, although their function in the allo-SCT setting has not been fully explored [28]. 

MSCs may be used for hematopoiesis enhancement, GVHD prophylaxis, and treatment of established severe acute GVHD in allo-HSCT patients. Previous studies have supported the use of MSCs in steroid-refractory GVHD [11,21,22,23,24,27]. Intravenous administration of MSCs has been well tolerated [29]. In 1994, Le Blanc et al. reported a case with successful treatment of grade IV acute GVHD of the gut and liver with third-party haploidentical MSCs. They postulated that MSCs have a potent immunosuppressive Veffect in vivo [30]. In 1998, the same researchers published the MSC treatment of steroid-resistant, severe, acute, and chronic GVHD as a phase II study [22]. Herrmann et al. reported a phase I study that was applied to MSC therapy for steroid-refractory acute and chronic GVHD. They administered 2 infusions per patient and the overall response rates for acute GVHD were complete in 7 and partial in 4, with no response in 1 patient. Of the 7 patients who achieved a complete response, 6 were still alive [31]. 

The combination of cyclosporine and short course of methotrexate is currently considered the standard prophylaxis of GVHD [32]. There is sufficient in vitro evidence to support the use of MSCs in the prevention and treatment of GVHD. However, it has been rarely reported that MSCs were very effective for GVHD prevention in vivo but not in the treatment of GVHD in the xenogeneic model of NOD/SCID mice. In addition, these studies reported no adverse events following the infusion of MSCs, making it possible to use these cells for prevention of acute GVHD [33,34]. Tisato et al. designed a study to address these questions in a xenogeneic model testing the ability of umbilical cord blood-derived MSCs to prevent and/or treat GVHD. They reported that MSCs of cord-blood origin are effective in the prevention but not the treatment of GVHD [33]. Another study depicted that MSCs suppress the lymphocyte proliferation in vitro but fail to prevent GVHD in mice (34). However, our study evaluated the clinical potential of MSCs for controlling GVHD in rats. According to our results, prophylactic in vivo use of MSCs was as effective as the standard prophylactic regimen in preventing GVHD. Furthermore, we did not observe any adverse events following the infusion of MSCs. Until recently, there were no published data regarding the preferred dose, timing, and frequency of MSC infusion. However, a randomized controlled phase III trial on the use of MSCs in acute GVHD in humans is currently underway, and the preliminary results are promising [35]. Recently, Kuzmina et al. reported a phase II human study on the use of MSCs for prevention of acute GVHD. This prospective clinical trial was based on random patient allocation to two groups receiving either standard GVHD prophylaxis or standard GVHD prophylaxis combined with MSC infusion. They demonstrated the efficacy of MSCs in GVHD prophylaxis in a limited number of patients with no adverse events directly attributable to administration of MSCs (36). However, our in vivo rat study displayed similar efficacies for both the standard GVHD prophylactic regimen (CsA+MTX) and the prophylactic MSC regimen alone. The immunosuppressive effect of combination of standard GVHD prophylaxis (CsA+MTX) and MSC infusion was also more potent. Although MSC treatment was tolerated well, this potent immunosuppressive effect of the CsA+MTX+MSC combination was associated with increased mortality in our study. The potent immunosuppressive effect of CsA+MTX+MSC combination is to be proven by further studies. 

To conclude, clinical use of MSCs in both prophylaxis against and treatment of established GVHD seems to be effective. However, MSC infusion combined with the standard GVHD prophylactic regimen causes stronger immunosuppression, with the potential of resultant early mortality. Clinical use of MSCs in prophylaxis and treatment of GVHD after allo-BMT requires further clinical trials.

## CONFLICT OF INTEREST STATEMENT

The authors of this paper have no conflicts of interest, including specific financial interests, relationships, and/ or affiliations relevant to the subject matter or materials included.

## Figures and Tables

**Table 1:**
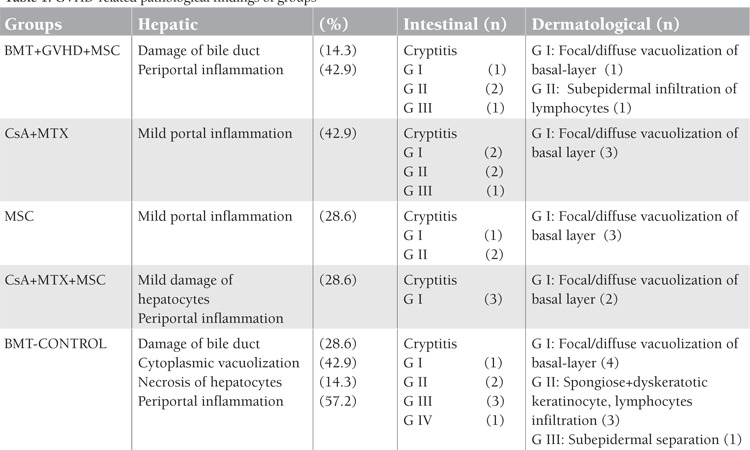
GVHD-related pathological findings of groups
